# Therapeutic Efficacy of Esomeprazole in Cotton Smoke-Induced Lung Injury Model

**DOI:** 10.3389/fphar.2017.00016

**Published:** 2017-01-26

**Authors:** Christina Nelson, Jameisha Lee, Kang Ko, Andrew G. Sikora, Mark D. Bonnen, Perenlei Enkhbaatar, Yohannes T. Ghebre

**Affiliations:** ^1^Department of Anesthesiology, University of Texas Medical BranchGalveston, TX, USA; ^2^Department of Otolaryngology-Head and Neck Surgery, Baylor College of MedicineHouston, TX, USA; ^3^Department of Radiation Oncology, Baylor College of MedicineHouston, TX, USA

**Keywords:** proton pump inhibitors, fibrosis, inflammation, lung injury

## Abstract

Proton pump inhibitors (PPIs) are well-known antacid drugs developed to treat gastric disorders. Emerging studies demonstrate that PPIs possess biological activities that extend beyond inhibition of H^+^/K^+^ ATPase (proton pumps) expressed in parietal cells of the stomach. Some of the extra-gastric activities of PPIs include modulation of epithelial, endothelial, and immune cell functions. Recently, we reported that PPIs suppress the expression of several proinflammatory and profibrotic molecules, as well as enhance antioxidant mechanisms in order to favorably regulate lung inflammation and fibrosis in an animal model of bleomycin-induced lung injury. In addition, several retrospective clinical studies report that the use of PPIs is associated with beneficial outcomes in chronic lung diseases including idiopathic pulmonary fibrosis (IPF) and chronic obstructive pulmonary disease (COPD). Based on these preclinical and clinical observations, we hypothesized that PPIs ameliorate smoke-induced lung injury. Accordingly, we evaluated the pharmacological efficacy of the PPI esomeprazole in a mouse model of cotton smoke-induced lung injury. The animals were exposed to cotton smoke for 3-weeks in the presence or absence of esomeprazole treatment. We found that therapeutic administration of esomeprazole significantly inhibited the progression of fibrosis throughout the lungs of the animals in this group compared to controls. In addition, esomeprazole also reduced circulating markers of inflammation and fibrosis. Overall, our work extends the emerging anti-inflammatory and antifibrotic potential of PPIs and their role in modulation of chronic lung diseases.

## Introduction

For nearly 30 years, the field of gastroenterology has been substantially impacted by the development and progressive use of proton pump inhibitors (PPIs) for the treatment of disorders characterized by overproduction of gastric acid including gastroesophageal reflux disease (GERD). In 2015, PPIs were among the 10 most prescribed and 10 most sold drugs in the United States (Brown, 2015). In addition, PPIs are among WHO's model list of essential medicines for adults and children (World Health Organization, 2015a,b).

However, little is known about possible extra-gastrointestinal role of PPIs in general and regulation of the pulmonary system in particular. Lately, *in vitro, in vivo* and retrospective clinical data has been emerging to suggest additional clinical utility of the PPIs in non-gastric diseases including cancer (Goh et al., 2014; Canitano et al., 2016; Fais, 2016), idiopathic pulmonary fibrosis (IPF; Raghu et al., 2006; Lee et al., 2011; Ghebremariam et al., 2015a), and chronic obstructive pulmonary disease (COPD; Sasaki et al., 2011). One aspect of PPIs that might be responsible for their pleiotropic pharmacological effect is the incorporation of a benzimidazole scaffold into their structure (Shin et al., 2004). Medicinal chemists and drug developers consider small molecules with benzimidazole cores as “privileged” in that the scaffold is able to simultaneously target several biological molecules (Bansal and Silakari, 2012; Gaba et al., 2014; Kaur et al., 2014). In fact, about 25% of the 100 best-selling drugs incorporate a benzimidazole moiety (Khokra and Choudhary, 2011).

In previous studies, we have applied biochemical, cellular, and animal models to characterize the effect of PPIs on alternate biological targets. First, we discovered that the entire class of PPIs directly inhibit the enzymatic activity of dimethylarginine dimethylaminohydrolase (DDAH) (Ghebremariam et al., 2013). Genetic and pharmacological studies show that DDAH is pathologically involved in the disease process in IPF (Pullamsetti et al., 2011). One mechanism by which DDAH is involved in IPF is through dysregulation of inducible nitric oxide synthase (iNOS) activity. Higher DDAH levels are expected to enhance the enzymatic degradation of asymmetric dimethylarginine (ADMA); the endogenous and competitive iNOS inhibitor (Ogawa et al., 1987). Thus, the brake placed by ADMA on iNOS can be released by overly active DDAH resulting in higher iNOS expression and/or activity. Overexpression of iNOS is associated with increased nitrosative stress and lung injury whereas its genetic knockout or pharmacological inhibition suppresses pathological lung remodeling in animal models of bleomycin- and smoke-induced lung injury (Genovese et al., 2005; Seimetz et al., 2011).

Using primary human lung fibroblasts, epithelial, and endothelial cells, we have shown that PPIs inhibit the expression of iNOS and other proinflammatory molecules including tumor necrosis factor alpha (TNFα), interleukins, and adhesion molecules in response to bleomycin treatment (Ghebremariam et al., 2015a). In addition to the regulation of the DDAH/iNOS pathway, we have also demonstrated that PPIs possess anti-proliferative and antifibrotic properties (Ghebremariam et al., 2015a; Ghebre Y. T. and Raghu, 2016). The later effect of PPIs might be associated with the upregulation of heme oxygenase 1 (HO1) that is observed upon treatment of these cells with PPIs (Becker et al., 2006; Ghebremariam et al., 2015a; Ghebre Y. and Raghu, 2016). Increased HO1 expression and/or activity is expected to unleash the protective effects of 3 major biological molecules that are released upon HO1-mediated catabolism of heme: carbon monoxide, bilirubin, and ferritin (Morse and Choi, 2002; Slebos et al., 2003). Several other *in vitro* studies also reported that PPIs have direct free-radical scavenging activities and inhibit the adhesion of inflammatory cells to vascular endothelial cells (Lapenna et al., 1996; Yoshida et al., 2000; Simon et al., 2006).

Our *in vivo* study in a rat model of bleomycin-induced lung injury demonstrated that the PPI esomeprazole significantly and favorably modulates lung inflammation and fibrosis resulting in reduced lung scarring and destruction of surfactant protein C (SPC) positive cells (Ghebremariam et al., 2015a).

In the present study, we assessed the prophylactic and therapeutic potential of esomeprazole in a mouse model of cotton smoke-induced lung injury. In addition, we evaluated plasma samples for circulating markers of inflammation and fibrosis.

## Materials and methods

### Cotton smoke-induced lung injury model

The *in vivo* study was performed following approval by the animal ethics committee of the University of Texas Medical Branch, Galveston and all ethical principles on the use of animals in research were adhered. We used 8-weeks old C57BL/6J mice (25–30 g body weight) to evaluate the efficacy of esomeprazole in a 3-weeks course of cotton smoke-induced lung injury; an established model that is useful to evaluate acute lung injury perpetrated by smoke from sources other than tobacco (Han et al., 2015). The number of animals per study group was calculated using Power and Sample Size Calculation (PS v3.1.2; Vanderbilt University) to achieve normally distributed data that has a standard deviation (σ) of 0.15 to detect a 20% difference in the means (δ) of vehicle and treatment groups at a significance (α) level of 0.05 and 80% power (β). Subsequently, all the animals were randomized into no exposure (*n* = 6) and exposure groups. Next, the exposure group of animals were subdivided into vehicle (*n* = 10), prophylactic esomeprazole (*n* = 10), and therapeutic esomeprazole (*n* = 10) and were subjected to cotton smoke for 21-days. Starting at 2 days post inhalation injury, the vehicle and prophylactic esomeprazole groups were orally treated daily with equal volume of 10% ethanol or 300 mg/kg esomeprazole in 10% ethanol, respectively. In the therapeutic esomeprazole group, the animals were treated daily starting from day 10 post-initiation of smoke exposure until necropsy. At necropsy, blood samples were collected by cardiac puncture and the lung, liver, heart, and kidney tissues were harvested for organ weight and histopathological comparisons.

### Biochemical studies

The blood samples were collected in EDTA containing tubes and plasma was separated by centrifugation at 5000 rpm for 15 min. Subsequently, the plasma samples were transferred into fresh tubes and frozen at −80°C for biochemical studies. The concentrations of ADMA, NO, TNFα, IL1β, and matrix metalloproteinase type 7 (MMP7) were determined by ELISA and respective standard curves were used for comparisons. For the ADMA study, frozen plasma samples were thawed and assayed as recommended by the manufacturer (DLD Diagnostika, Hamburg, Germany). In brief, 20 μL of diluted plasma from each group was transferred into a 96-well plate for ADMA acylation prior to overnight incubation of the acylated ADMA with rabbit anti-acyl-ADMA Antibody. Subsequently, unbound analytes were washed off the plate and TMB substrate was used to develop the reaction. The concentration of ADMA in the samples was calculated from a standard curve obtained by measuring absorbance of the provided standards at 450 nm as described (Schulze et al., 2004; Ghebremariam et al., 2015b). For the NO, TNFα, IL1β, and MMP7 studies, kits were purchased from Assay Designs (Ann Arbor, MI), Invitrogen (Camarillo, CA), Sigma (St Louis, MO), and Kamiya Biomedical Company (Seattle, WA), respectively. Total plasma NO was measured in the form of nitrite using a colorimetric assay that allows reduction of nitrate into nitrite prior to quantification. For TNFα, IL1β, and MMP7 assays, antibody directed at the respective analyte was coated onto the wells of microtiter strips and diluted plasma, controls, and standards were allowed to react to the capture antibody. Biotin and streptavidin were used to develop the reaction. The intensity of the color produced, proportional to the concentration of analyte present in the samples, was measured using a plate reader at 450 nm. The mean value of each data was used to compare the groups.

### Histopathological studies

Lung tissues were fixed in 10% formalin prior to cutting, embedding and staining with H&E and Masson's Trichrome for evaluation of inflammation and fibrosis respectively. The stained slides were evaluated microscopically by a board-certified pathologist who was blinded to the treatment that the animals received. For the H&E stained, multiple fields per slide were semi-quantitatively scored for inflammation on a scale of 1+ to 4+: 1+ = minimal change containing one or a few small foci in the alveoli and/or around the conducting airways; 2+ = mild change containing small to medium sized foci; 3+ = moderate change containing frequent and medium sized foci; and 4+ = marked change characterized by extensive and confluent foci affecting most of the tissue. For the Masson's Trichrome stained slides, fibrosis was scored based on a published and widely accepted system (Ashcroft et al., 1988) where grade 0 indicated essentially normal lung without fibrosis; and grade 8 represented complete fibrous obliteration of the normal lung architecture. All fields of an entire lung section per slide were scored individually and the mean score values were calculated for comparison.

### Statistical analysis

The number of animals per study group was calculated using Power and Sample Size Calculation as described above. GraphPad Prism (La Jolla, CA) was used in all the statistical tests post-necropsy. For the organ weight study, as well as for comparison of the concentrations of plasma ADMA, NO, TNFα, IL1β, and MMP7, mean value ± standard error of the mean (SEM) is shown. Multiple groups were compared using one-way analysis of variance (ANOVA) and Bonferroni tests. Pairwise comparisons were performed using student's *t*-test. For the survival study, Kaplan–Meier analysis was performed to establish the number of surviving animals per group over time. In each of the studies above, data was considered to be statistically significant when the *p*-value was below 0.05 (*p* < 0.05).

## Results

### Esomeprazole increases plasma ADMA and reduces NO

**Figure 1:**
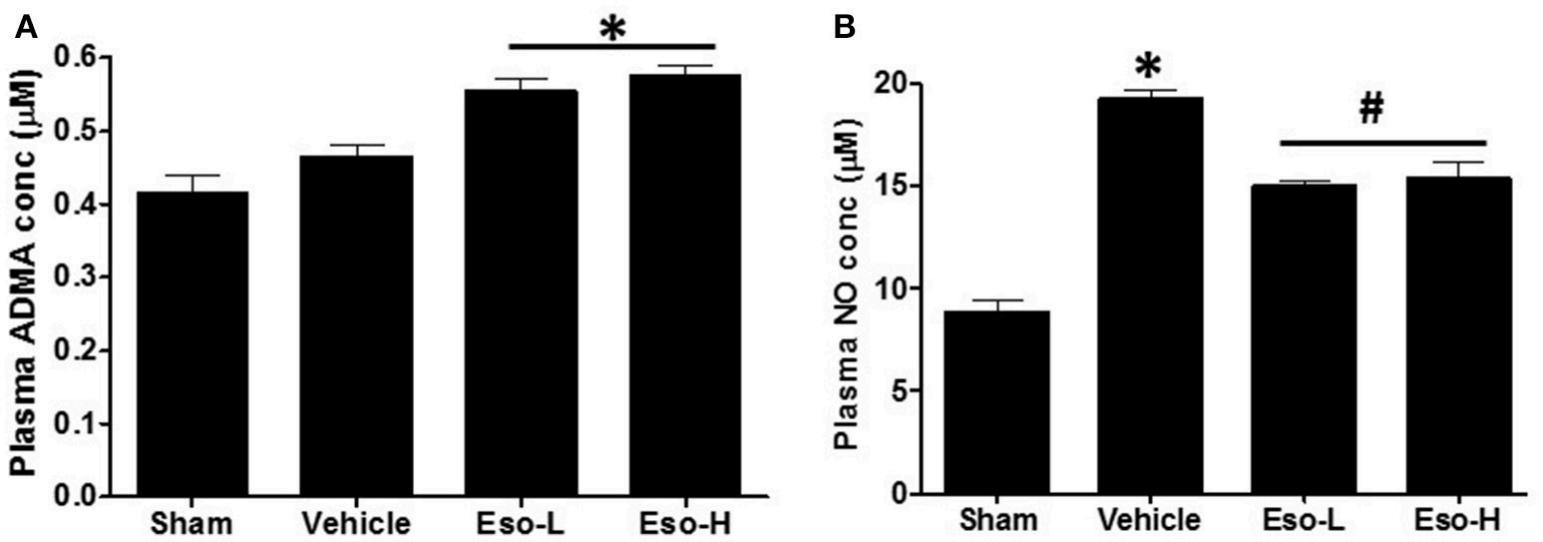
**Measurement of plasma ADMA and NO concentration at necropsy in sham (clean air), vehicle, and esomeprazole treated mice**. Exposure to smoke increased ADMA (sham vs. vehicle) and NO while esomeprazole treatment further spiked the concentration of ADMA (^*^p < 0.05 vs. sham group) to modulate NO (^*^p < 0.05 vehicle vs. sham group; ^#^*p* < 0.05 vehicle vs. Eso-L/Eso-H groups). Data is mean ± SEM from at least 5 animals per group run in replicates. Eso-L, low dose of esomeprazole (i.e. 30 mg/kg); Eso-H, high dose of esomeprazole (i.e., 300 mg/kg).

Lower DDAH enzymatic activity *in vivo* is reflected by higher concentration of ADMA and reduced NO in plasma and/or tissue (Palm et al., 2007; Lambden et al., 2015). Animals exposed to smoke showed higher levels of ADMA and NO suggesting reduction in DDAH activity and induction of iNOS. Moreover, the animals that were treated with esomeprazole showed higher plasma concentration of ADMA (Figure 1A) and reduction of NO (Figure 1B) compared to controls suggesting further inhibition of DDAH and iNOS *in vivo* by PPIs despite the effects of smoke on the enzymes. Recently, we have reported direct inhibition of DDAH enzymatic activity and reduction of iNOS by PPIs (Ghebremariam et al., 2013, 2015a).

### Esomeprazole attenuates circulating markers of inflammation and fibrosis

**Figure 2:**
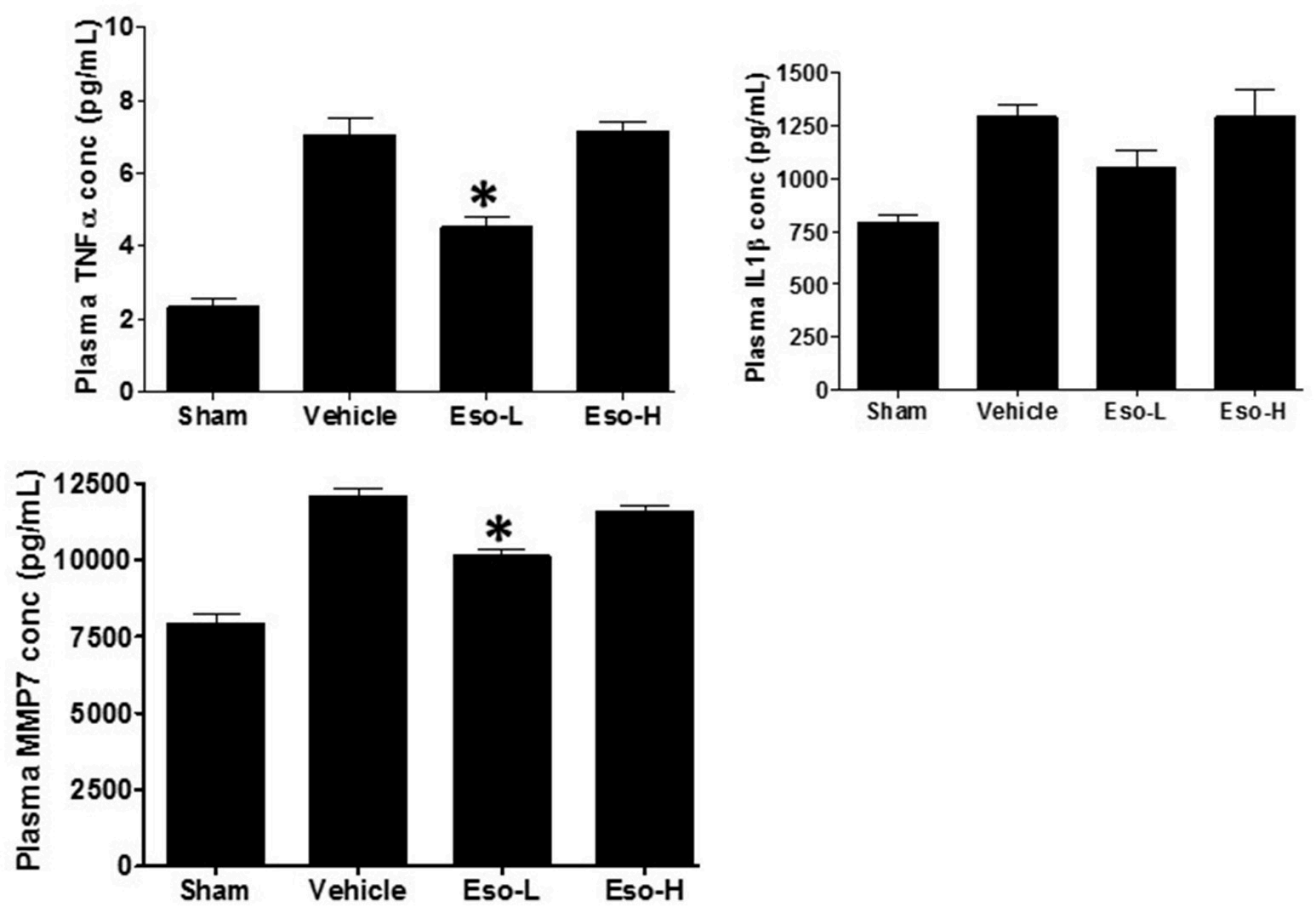
**ELISA-based measurement of circulating TNFα, IL1β, and MMP7 concentration in the plasma of sham, vehicle, and esomeprazole treated animals**. Smoke exposure increased levels of the proinflammatory cytokines (TNFα and IL1β), and the profibrotic protein MMP7 [sham (clean air) vs. vehicle]. However, therapeutic dose of esomeprazole reduced their concentration (^*^p < 0.05 vs. vehicle control). Data is mean ± SEM from at least 5 animals per group run in duplicates. Eso-L, low dose of esomeprazole; Eso-H, high dose of esomeprazole.

Exposure to tobacco or cotton smoke is known to induce proinflammatory cytokines including TNFα and interleukins in cultured cells *in vitro* and in animal models *in vivo* (Orosz et al., 2007; Lee et al., 2012). In our study, the levels of circulating TNFα and MMP7 were significantly lower in the low dose therapeutic esomeprazole group compared to vehicle or high dose prophylactic groups (Figure 2). In addition, the circulating level of IL1β showed a lower trend in the therapeutic esomeprazole group compared to the other experimental groups.

### Esomeprazole suppresses lung fibrosis

**Figure 3:**
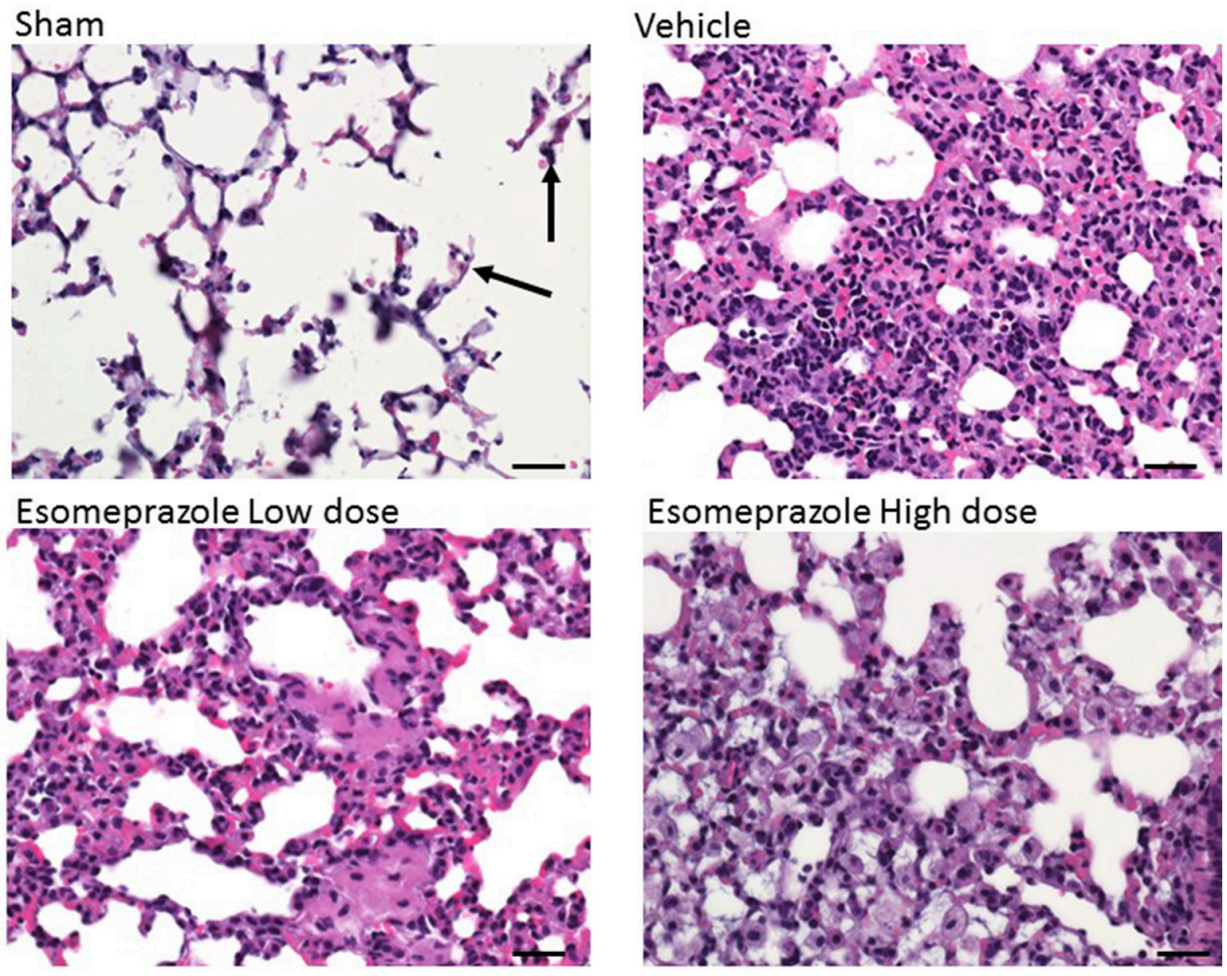
**H&E stained lung sections showing the presence of foamy macrophages and mixed cell infiltrates in animals exposed to cotton smoke and treated with vehicle or esomeprazole**. The sham (clean air) group shows normal lung architecture with a few alveolar macrophages (arrows). Image is at 40x magnification. Scale bar is 50 μm.

**Figure 4:**
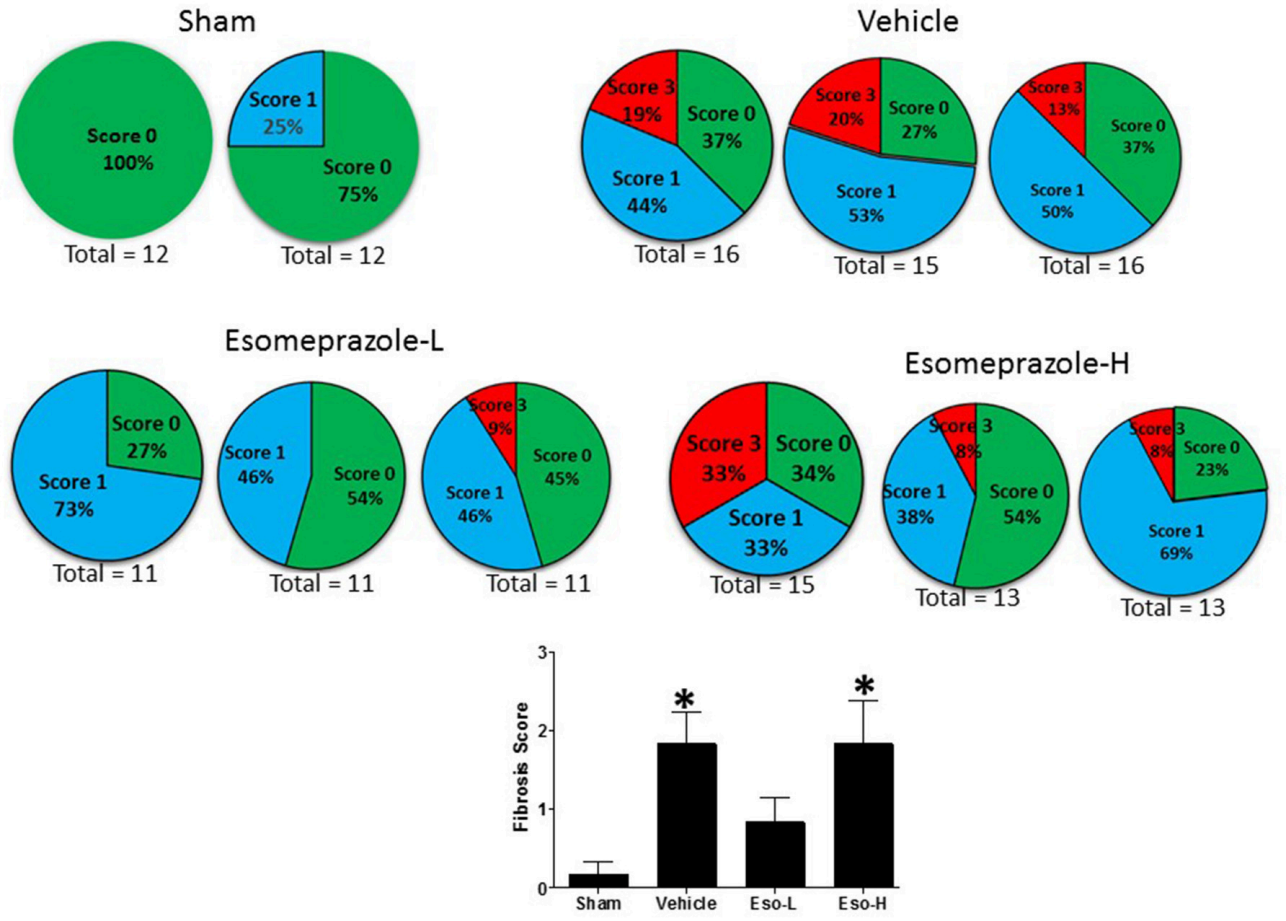
**Fibrosis score based on scanning of multiple non-overlapping fields of Masson's Trichrome stained sections of lung tissue harvested from animals exposed to clean air (sham) or cotton smoke**. The clean air exposed animals in the sham group show no fibrotic changes. However, the animals in the vehicle treated control group show higher fibrotic changes (indicated by red pie) while the animals in the therapeutic esomeprazole (Esomeprazole-L) group show normalized levels of collagen (indicated by blue pie) compared to vehicle or high-dose (Esomeprazole-H) group. Two or three slides per group were scanned and the total number of non-overlapping fields counted is shown below each pie. The averaged fibrotic score is shown as bar graph in the lower panel (^*^p < 0.05 vs. sham group).

**Figure 5:**
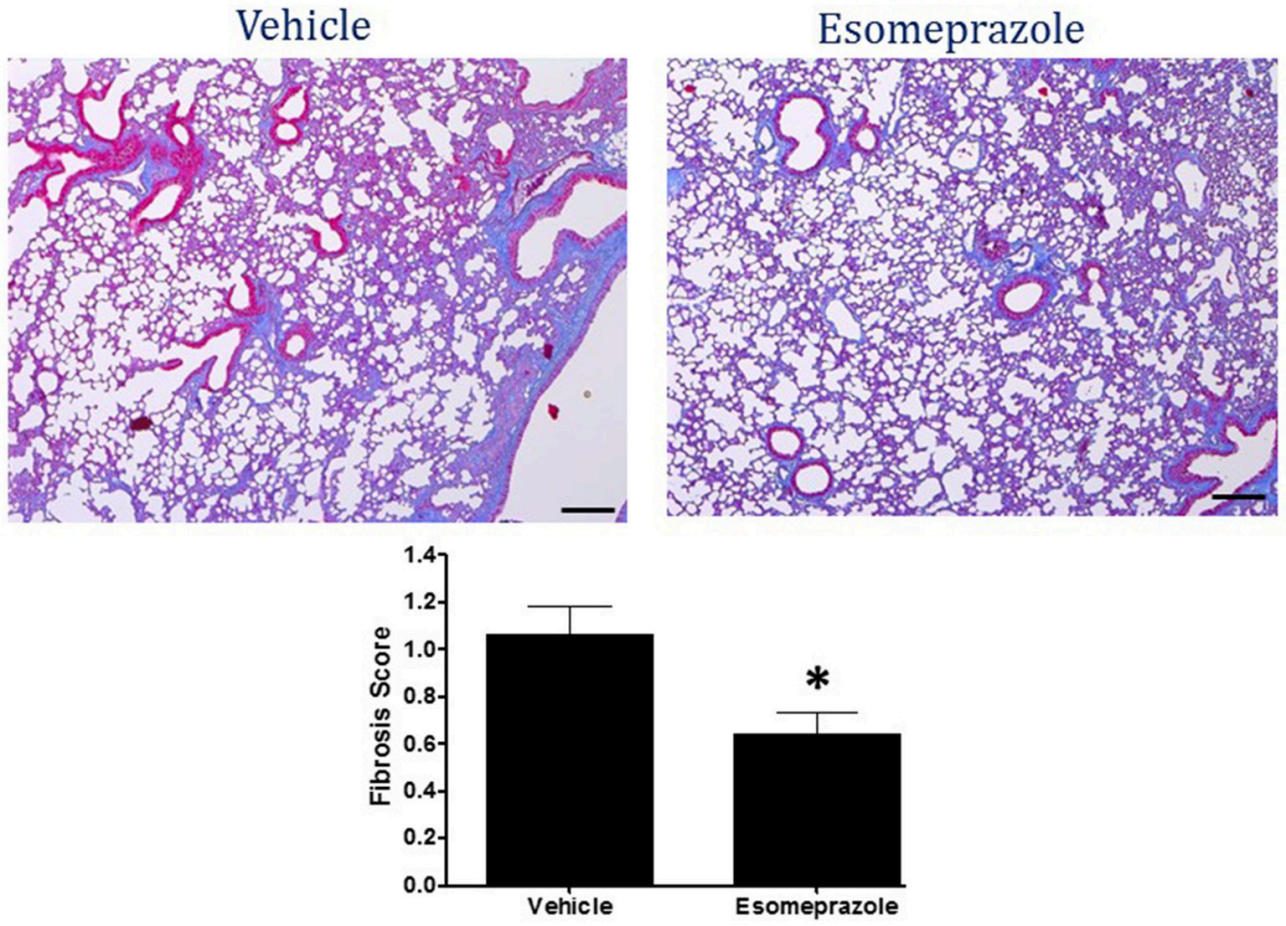
**Masson's Trichrome stain of lung tissue harvested from mouse model of smoke-induced lung injury**. Therapeutic esomeprazole (30 mg/kg) reduced collagen accumulation resulting in lower fibrosis score; lower panel ^*^p < 0.05). Image is at 40X objective magnification.

Smoke inhalation can induce and exacerbate severe organ damage including lung inflammation in preclinical models and human subjects (Murakami et al., 2002; Gualano et al., 2008; Lee et al., 2012). As an interface between the environment and the body, the lungs are particularly more susceptible to the effects of smoke whether released from cigarette or other combustible substance like burning of wood, paper, or cotton. In this study, the cotton smoke exposed animals had substantially increased number of foamy macrophages in the alveoli and focal inflammation around conducting airways compared to the animals exposed to clean air (Figure 3). Treatment with esomeprazole did not have favorable effect on lung inflammation in this model. For the fibrosis score, 10 or more non-overlapping fields were analyzed per lung section. Intriguingly, almost all the fields in the therapeutic esomeprazole group had no-to-minimal fibrosis score (between 0 and 1) while 64% of the fields in the vehicle group scored between 1 (i.e., minimal fibrous thickening of the alveoli) and 3 (i.e., moderate thickening of the alveolar walls) of which 17% had the histological feature of grade 3 fibrosis (Figure 4) indicating that the therapeutically administered low dose of esomeprazole inhibited fibrosis throughout the lungs. As described below, the high dose esomeprazole was not well tolerated by the animals resulting in increased mortality and no meaningful impact in suppressing lung fibrosis (Figure 4). For the low dose therapeutic esomeprazole group, representative histology demonstrating its efficacy is presented as Figure 5 (mean fibrosis score of 0.64 vs. 1.06 in the vehicle; *p* < 0.05).

### Esomeprazole treatment did not change organ weight

Recordings of total body and organ weight at necropsy showed that treatment with esomeprazole did not significantly alter the weight of the lungs, heart, and kidneys. However, there was a slight but insignificant increase in the weight of the liver in the esomeprazole group compared to vehicle (Figure S1). In addition, the animals in the high dose esomeprazole group (300 mg/kg) had increased incidences of bloating up, sluggishness, and mortality (Figure S2). Extensive preclinical and clinical studies have been conducted to test the tolerability, safety, and efficacy of PPIs when administered at various doses systemically. These studies have established that although side effects including liver toxicity may be seen at lower doses of PPI, the lethal dose in rodents is generally above 1 g/kg body weight.

## Discussion

Several studies have demonstrated that the DDAH/iNOS pathway is pathologically involved in a number of pulmonary disorders including asthma (Trifilieff et al., 2000; Batra et al., 2007), IPF (Pullamsetti et al., 2011), and COPD (Seimetz et al., 2011). Using a murine model of bleomycin-induced lung injury, Pullamsetti et al. (2011) showed that overexpression of DDAH plays a pathological role in lung inflammation and fibrosis at preclinical and clinical levels. This study also revealed that iNOS and DDAH co-localize more densely in the alveoli of IPF-derived lung sections compared to lungs obtained from healthy donors.

Given the significance of iNOS/DDAH involvement in lung pathobiology and predicted druggability of this pathway, it is logical to test and develop small molecule inhibitors of these enzymes. Accordingly, we screened over 100,000 small molecules against the DDAH enzyme and reported several small molecules as DDAH inhibitors (Ghebremariam et al., 2012, 2014). Intriguingly, we discovered that the entire class of PPIs inhibits the enzymatic activity of DDAH (Ghebremariam et al., 2013). Subsequent studies revealed that PPIs downregulate the expression of iNOS and several other proinflammatory cytokines (Ghebremariam et al., 2015a). Our *in vivo* study also demonstrated that a prototype PPI, esomeprazole, attenuates bleomycin-induced lung inflammation and fibrosis and surfaced the idea of PPIs as potential antifibrotic drugs (Ghebre Y. T. and Raghu, 2016).

In the present study, we extended the assessment of esomeprazole's emerging pharmacological role in airway diseases into cotton smoke-induced lung injury and remodeling. This animal model recapitulates many features of acute lung injury including oxidative stress, upregulation of iNOS expression and activity, as well as inflammatory and fibrotic changes (Enkhbaatar, 2015; Han et al., 2015). Although, our initial intention was to evaluate the prophylactic and therapeutic potential of esomeprazole in a mouse model of smoke-induced lung injury at a dose of 300 mg/kg, the animals in the prophylactic group (which started esomeprazole treatment 2 days post-initiation of smoke insufflation), were increasingly unable to tolerate this dose of esomeprazole and started dropping out before the pre-scheduled therapeutic arm could begin treatment. The bloating up, sluggishness, and mortality seen in the prophylactic group compelled us to lower the dose of the therapeutic arm. By contrast to the effects seen in mice, our study of high dose esomeprazole (300 mg/kg) in rats was well tolerated and showed significant effect on lung inflammation and fibrosis (Ghebremariam et al., 2015a). In the present study, however, the therapeutic esomeprazole group which received 30 mg/kg esomeprazole treatment 10 days post-initiation of smoke injury, showed good tolerance of the drug and therapeutic benefits including inhibition of DDAH enzymatic activity, suppression of circulating proinflammatory and profibrotic markers, as well as attenuation of lung tissue scarring (Figure 5). Despite the suppression of systemic inflammation, the lack of significant inhibition of inflammation in the lungs is not clear but indicative of reduced bioavailability of the drug in the lung tissue itself.

Several cohorts of retrospective studies have reported that IPF patients on “antacid” therapy (>85% of PPIs) have favorable respiratory outcomes including greater diffusing capacity of the lung for carbon monoxide (DL_CO_), prolonged median transplant-free survival time, fewer to no episodes of acute exacerbations, and fewer IPF-related, as well as all-cause mortality (Raghu et al., 2006; Lee et al., 2011, 2013; Noth et al., 2012; Ghebremariam et al., 2015a; Ghebre, 2016; Kreuter et al., 2016). Recently, the American Thoracic Society (ATS) and its sister respiratory societies in Europe (ERS), Japan (JRS), and Latin America (ALAT) released an official statement that includes the conditional recommendation for the treatment of IPF patients with PPIs regardless of the GERD status of patients (Raghu et al., 2011). Similar to the potentially beneficial effects of PPIs in IPF, a prospective study in COPD patients also reported such beneficial outcomes associated with PPI use (Sasaki et al., 2009, 2011). Based on ours and several other reports, it is becoming evident that PPIs have biological functions that extend beyond mere reduction of gastric acidity (Ghebre Y. and Raghu, 2016). The growing number of extra-intestinal diseases for which PPIs may have clinical utility include respiratory diseases. However, parallel to the demonstration of biological efficacy, it is important to dissect the mechanistic basis by which PPIs may regulate non-gastric cells. Our previous cell biological and molecular studies have uncovered polypharmacological properties of PPIs by showing downregulation of several classic proinflammatory molecules and upregulation of HO1. Our unpublished data indicates that the later effect of PPI on lung cells is due to regulation of the Keap1/Nrf2 pathway. This effect of PPIs is consistent with compounds that harbor electrophile moieties in their structure and trigger chemical stress on cellular processes to ultimately induce cytoprotective mechanisms (Satoh et al., 2013). Such mechanistic understanding becomes particularly important for PPIs due to long standing comorbid confounders such as GERD in respiratory diseases including IPF and COPD. Although, GERD might be involved in IPF/COPD disease pathogenesis through reflux and microaspiration, PPIs neither prevent reflux nor block microaspiration of acidic droplets (Raghu, 2011; Ghebre Y. T. and Raghu, 2016). Thus, it appears that PPIs may be able to regulate lung injury without having therapeutic efficacy on GERD.

In conclusion, it is important to realize that PPIs are administered as prodrugs in the treatment of GERD. Once in the stomach, the acidic microenvironment enforces chemical rearrangement of the prodrug and eventual transformation into sulfenic acids or sulfonamide analogs to block the H^+^/K^+^ pumps that are expressed in parietal cells (Shin and Sachs, 2008). However, our cell biological and molecular studies are conducted at neutral to basic pH (7.4–7.6) with freshly prepared drug (Ghebremariam et al., 2015a). Although, exposure to low pH (<5) triggers rapid degradation of the PPI prodrug into these derivatives, there is no appreciable conversion of the prodrug into sulfenic acids or sulfonamides at higher pH (>6.1; Shin et al., 2004). In addition, the present study was conducted in mice; specie that do not have reflux response physiologically. Thus, our findings, in light of these accounts, argue that the prodrug in GERD may actually be the active one in the *direct* modulation of processes involved in lung inflammation and fibrosis.

## Author contributions

Participated in research design: YG, PE; Conducted experiments: CN, JL, KK, YG; Performed data analysis: CN, YG, PE; Wrote or contributed to the writing of the manuscript: YG, CN, PE, AS, MB.

### Conflict of interest statement

YG is an inventor on a patent, owned by Stanford University, that protect the development of PPIs for therapeutic use of new indications including lung injury. YG is a cofounder of Altitude Pharma, Inc.; a biotechnology company pursuing PPI-based therapy for lung fibrosis. The other authors declare that the research was conducted in the absence of any commercial or financial relationships that could be construed as a potential conflict of interest.

## Supplementary material

Figure S1 **Measurement of organ weight at necropsy following smoke-induced lung injury in a mouse model**. There was no difference in the weight of the lungs, heart and kidneys. However, the liver was slightly enlarged in the esomeprazole treated groups (*p* > 0.05). Data is mean ± SEM from at least 5 animals per group.

Figure S2 **Kaplan-Meier survival plot of mice exposed to clean air (sham) or cotton smoke and treated with vehicle (10% ethanol) or esomeprazole at low (30 mg/kg; Esomeprazole-L) or high (300 mg/kg; Esomeprazole-H) concentration**. There was increased mortality rate in the animals treated with high esomeprazole dose.
