# Supplementary figures and images for: Therapeutic Efficacy of Esomeprazole in Cotton Smoke-Induced Lung Injury Model
Source: Front Pharmacol. 2017 Jan 26;8:16. doi: 10.3389/fphar.2017.00016 (PMC5266706; doi:10.3389/fphar.2017.00016)

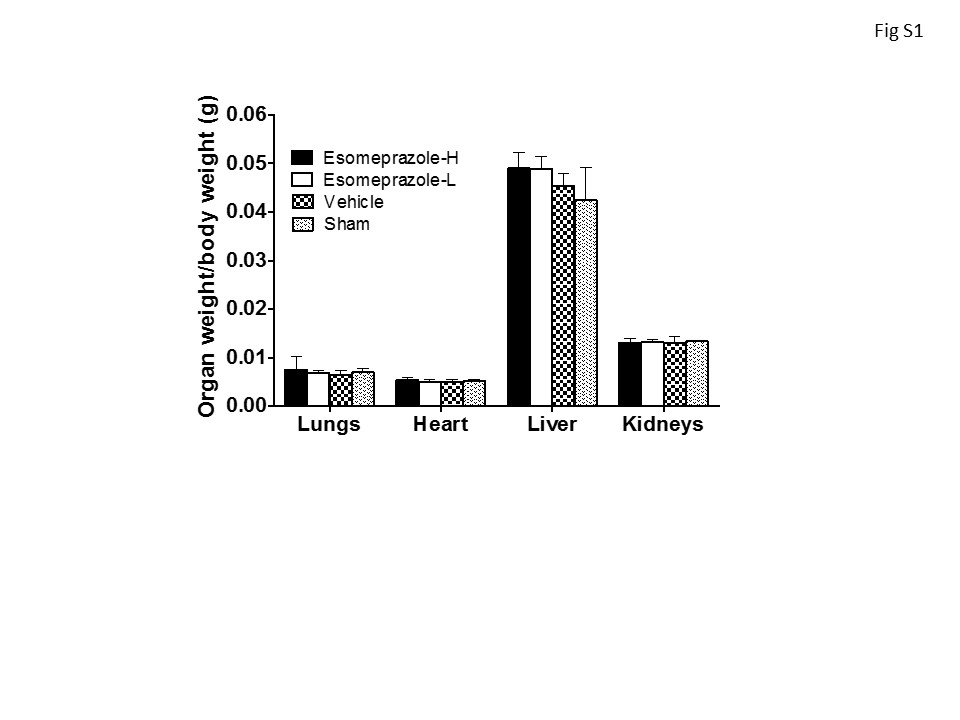

Supplement: Figure S1 — Measurement of organ weight at necropsy following smoke-induced lung injury in a mouse model. There was no difference in the weight of the lungs, heart and kidneys. However, the liver was slightly enlarged in the esomeprazole treated groups (p > 0.05). Data is mean ± SEM from at least 5 animals per group. [file Image1.JPEG]

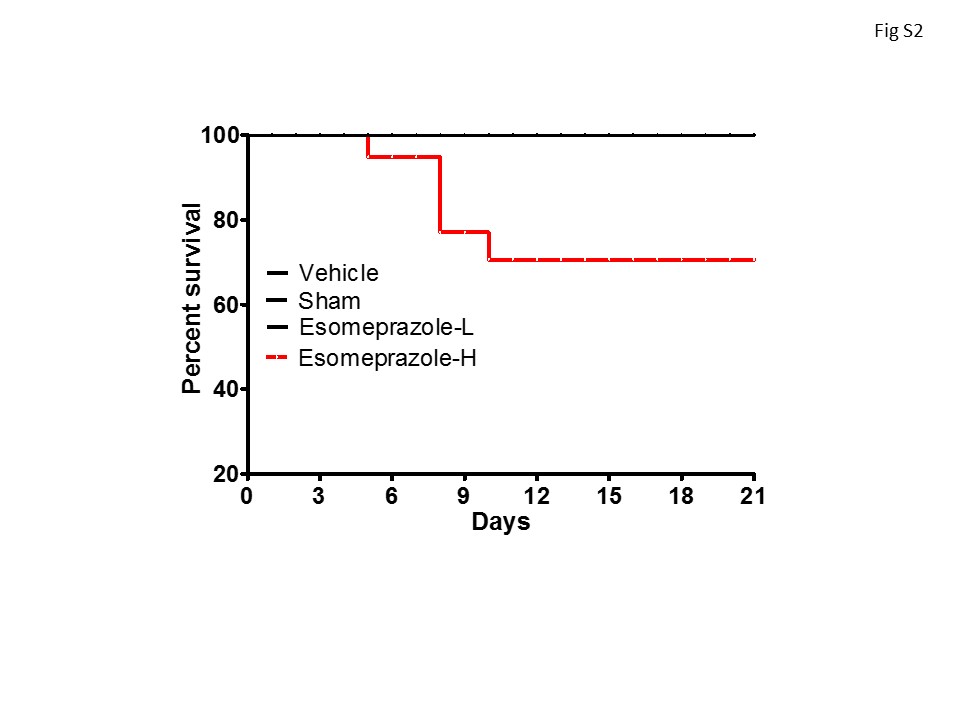

Supplement: Figure S2 — Kaplan-Meier survival plot of mice exposed to clean air (sham) or cotton smoke and treated with vehicle (10% ethanol) or esomeprazole at low (30 mg/kg; Esomeprazole-L) or high (300 mg/kg; Esomeprazole-H) concentration. There was increased mortality rate in the animals treated with high esomeprazole dose. [file Image2.JPEG]
